# Spontaneous rupture of an unscarred uterus during pregnancy: A rare but life-threatening emergency: Case series

**DOI:** 10.1097/MD.0000000000033977

**Published:** 2023-06-16

**Authors:** Yue Chen, Ying Cao, Jing-Yao She, Si Chen, Pei-Juan Wang, Zheng Zeng, Chun-Yun Liang

**Affiliations:** a The Third Clinical Medical College, Nanjing University of Chinese Medicine, Nanjing, China; b Department of Obstetrics and Gynecology, Affiliated Hospital of Integrated Traditional Chinese and Western Medicine, Nanjing University of Chinese Medicine, Nanjing Jiangsu, China; c Department of Obstetrics and Gynecology, Jiangsu Province Academy of Traditional Chinese Medicine, Nanjing, China; d Department of Obstetrics and Gynecology, Suzhou Hospital of Traditional Chinese Medicine Affiliated to Nanjing University of Chinese Medicine, Suzhou, China; e Department of Pathology, Jiangsu Province Academy of Traditional Chinese Medicine, Nanjing, China.

**Keywords:** placenta accreta, pregnancy complications, risk factor, scarless, uterine rupture

## Abstract

In most cases, uterine rupture occurs during the third trimester of pregnancy or during labor. Even fewer reports have been published about the occurrence of this condition without a gynecologic history of any surgical procedure. Due to their scarcity and variable clinical presentation, early diagnosis of uterine rupture may be difficult, and if the diagnosis is not timely, the condition may be life-threatening.

**Patient concerns:**

Herein, 3 cases of uterine rupture from a single institution are described. Three patients are at different gestational weeks and all have no history of uterine surgery. They came to the hospital due to acute abdominal pain, which is characterized by severe and persistent pain in the abdomen, with no apparent vaginal bleeding.

**Diagnoses:**

All 3 patients were diagnosed with uterine rupture during the operation.

**Interventions:**

One patient underwent uterine repair surgery; while the other 2 underwent subtotal hysterectomy due to persistent bleeding and pathological examination after surgery confirmed placenta implantation.

**Outcomes:**

The patients recovered well after the operation, and no discomfort occurred in the follow-up.

**Lessons:**

Acute abdominal pain during pregnancy can pose both diagnostic and therapeutic challenges. It is important to consider the possibility of uterine rupture, even in cases where there is no history of prior uterine surgery. The key to the treatment of uterine rupture is to shorten the diagnosis time as much as possible, this potential complication should be carefully monitored for and promptly addressed to ensure the best possible outcomes for both the mother and the developing fetus.

## 1. Introduction

There are severe maternal and infant complications associated with uterine rupture during pregnancy, which can be life-threatening if not detected in a timely manner. Unscarred uterine rupture is an extremely rare emergency, while women and their newborns are at a higher risk of mortality and morbidity.^[1]^ Despite the fear of this complication, there is currently no clinical tool to predict its development.^[2–4]^ Clinical diagnosis of uterine rupture is challenging due to the broad variation in symptoms and progress. Imaging diagnostics might not be able to quickly and effectively identify uterine rupture, particularly in its early stages. In all pregnant women presenting with an acute abdomen, regardless of medical history and different trimesters, the likelihood of uterine rupture should be considered, and early surgical intervention will result in a better prognosis. Here, we describe 3 cases of spontaneous uterine rupture occurring during pregnancy without any apparent probable risk factors, stressing the importance of awareness and early diagnosis and management of this rare complication.

## 2. Case presentation

Three cases of uterine rupture were identified in the Department of Obstetrics and Gynecology (Affiliated Hospital of Nanjing University of Chinese Medicine, Nanjing, China) between 2018 and 2020. In all cases, uterine rupture was confirmed by operation. Affiliated Hospital of Nanjing University of Chinese Medicine’s Institutional Review Board ethics committee reviewed and approved the study. These cases highlight the broad spectrum of presenting symptoms, pathologic features, and treatment outcomes associated with uterine rupture.

## 3. Case 1

**Figure 1.**
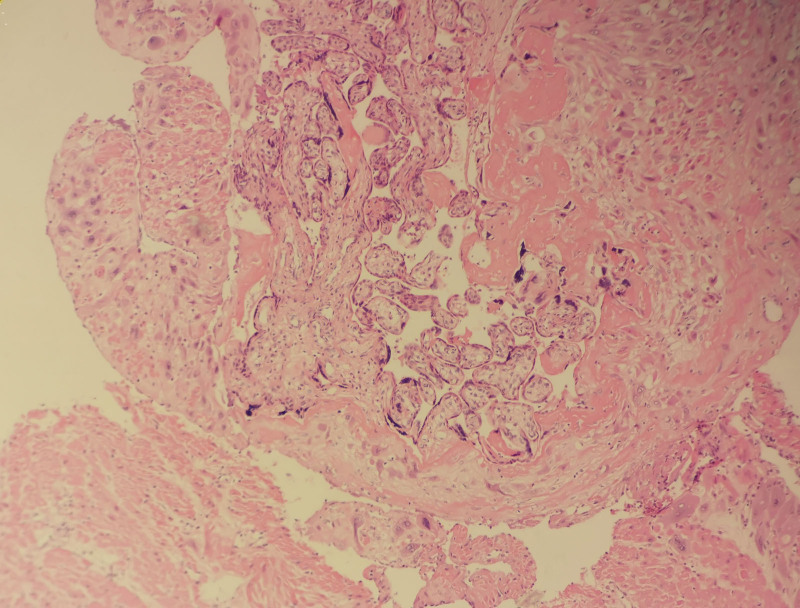
Postoperative pathologic examination of the operative specimen confirmed the presence of placenta accreta (H&E stain, 100 × magnification).

A 36-year-old woman who was at week 30 of gestation presented at the emergency department with aggravating epigastric and right lower abdominal pain for 6 hours. The pain was continuous with frequent exacerbation and was associated with nausea and vomiting. Her heart rate was 103 per minute and no hypotension was found. An abdominal examination revealed distention, tenderness, rebound pain, and shifting dullness positive. Emergency bedside urologic and obstetric ultrasound were negative, while the abdomen ultrasound showed a large amount of perihepatic effusion. The patient’s obstetric history included 2 ectopic pregnancies (once underwent a laparoscopic unilateral salpingectomy in 2008, once received conservative treatment in 2014) and 2 induced abortions. Based on the results described above, primary liver rupture was the first consideration. The patient rapidly developed shock after admission, immediately abdominal paracentesis under ultrasound guidance was conducted and uncoagulable blood was drained out; thus, emergent laparotomy was performed by the general surgeon. Exploratory laparotomy revealed massive hemoperitoneum (3500 mL). The active bleeding source was located in the anterior wall of uterusand, with a large irregular breach about 10 cm long. The fetus was still located in the uterus, but without signs of life when delivered. The placenta was dense adherent to the uterine wall without separation, placenta increta was considered at that time and a subtotal hysterectomy was performed. Postoperatively, the patient was transferred to the intensive care unit, who received massive blood transfusions and was monitored for 2 days. After this period of time, she was transferred to a general ward and discharged after 7 days. Pathological examination revealed placenta accreta (Fig. 1).

## 4. Case 2

**Figure 2.**
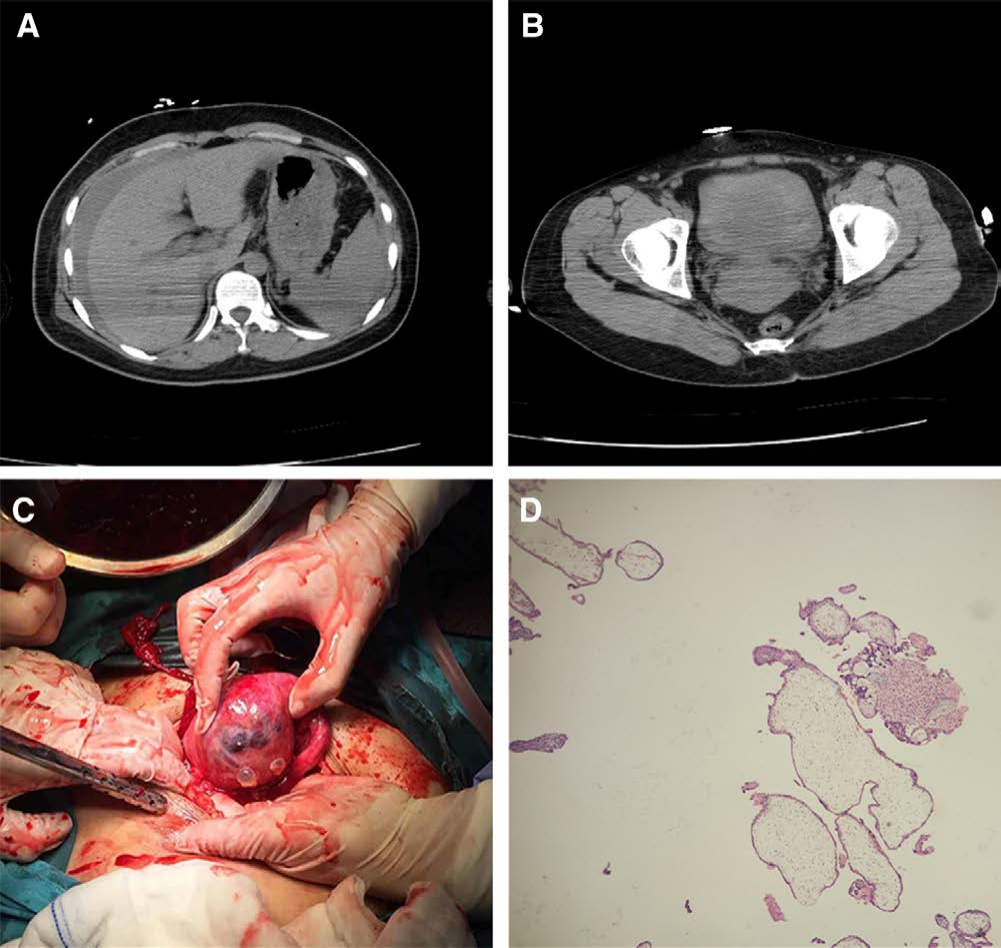
(A) Abdominal CT scan, massive perihepatic fluid collection (white arrow), (B) the enlarged uterus without evidence of rupture (white arrow), (C) uterine rupture: partial expulsion of the gestational sac out of the uterus (white arrow) (D) histology images show normal villi (H&E stain, 40x magnification). CT = computed tomography.

**Figure 3.**
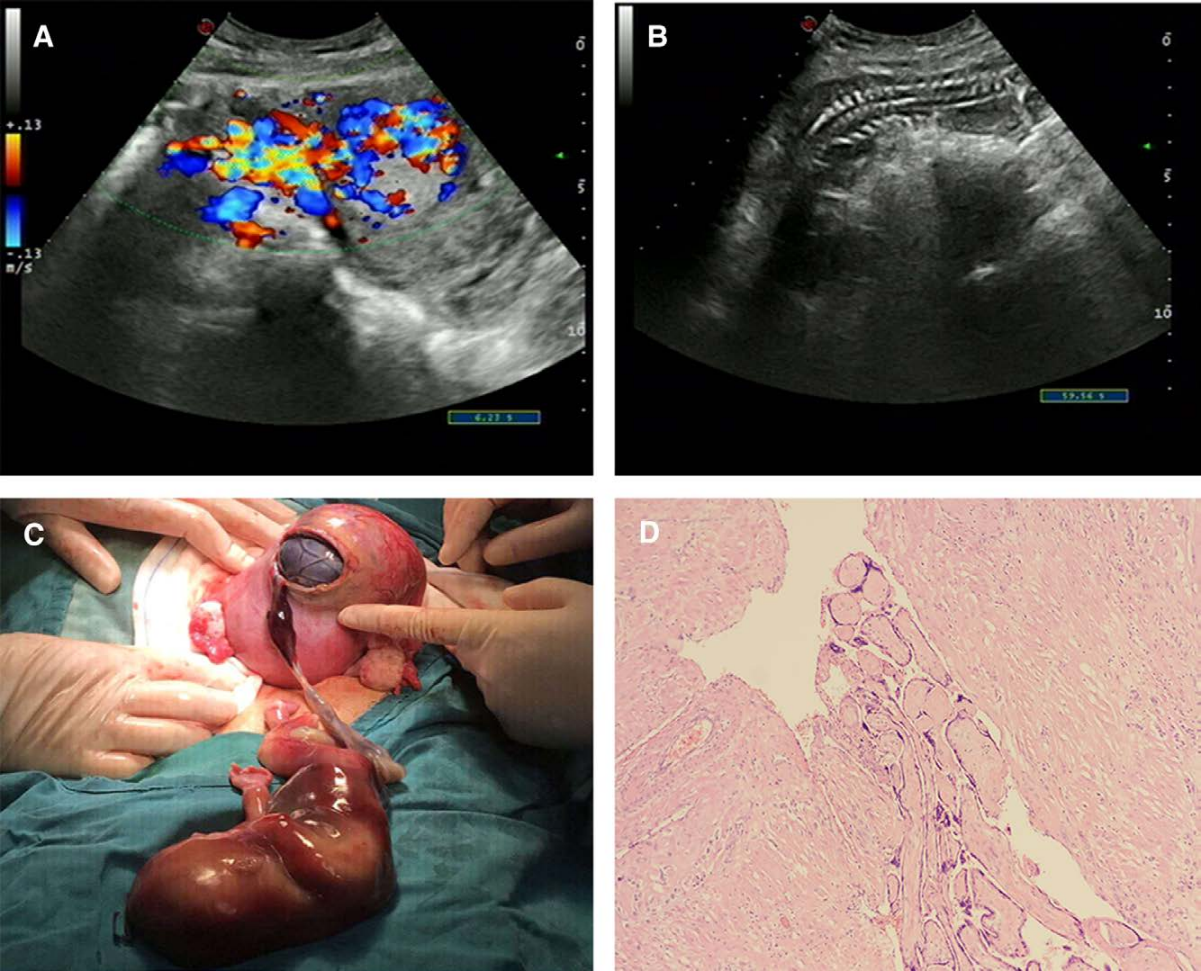
(A) Color doppler flow image showing the presence of altered placental blood flow (white arrow), (B) extra uterine fetuses (white arrow), (C) full thickness uterine rupture of 6 cm (white arrow) and a dead fetus in abdominal cavity with a placenta presenting sign of placental insertion, (D) histological examination: chorionic villi invaded the myometrium of the uterus (white arrow: villous tissue, black triangles: myometrium).

A 31-year-old woman with a spontaneous uterine rupture at 12st week of pregnancy without any identifiable potential risk factors. Her obstetric history was composed of 2 full-term vaginal deliveries. This was her third pregnancy. The pregnancy was conceived naturally, and there were no accidents until the 12-week gestational age. Patients presented to our emergency department with severe abdominal distension and pain lasting 1 hour, as well as dizziness and palpitations. The patient’s laboratory tests and vital signs showed no significant abnormalities. The body temperature was 37.0, the pulse was 68 beats/minutes, the respiration was 18 beats/minutes, the blood pressure was 150/94 mm Hg. On physical examination, the patient’s face was painful, the abdomen was slightly swollen, the lower abdomen was obviously tender, and there was suspicious shifting dullness. Emergency computed tomography scan of the whole abdomen showed abdominal effusion and an enlarged uterus, which was not in a position to diagnose the uterine rupture (Fig. 2A and B). Due to the patient’s pain continuing to worsen without relief and the diagnosis was not certain, an emergency laparoscopy was performed. Intraoperatively, massive hemoperitoneum was found but no obvious laceration in the liver and spleen during the examination. Subsequently, 2 breaches have been identified (0.7cm and 0.5cm) in the right posterior uterine with protrusion of the pregnancy sac (Fig. 3C). Uterine rupture was diagnosed and exploratory laparotomy was performed instead. A continuous suture was used to close the rupture of the uterus after the gestational sac was removed. Total bleeding was 4000 mL, and a total of 12U of suspended red blood cells, 1475 mL of plasma and 10u of cryoprecipitate were transfused during and after surgery. After the operation, the patient recovered and was discharged 8 days later. The patient recovered well after surgery. Histopathological examination was determined to be placental villi, but no other abnormalities were noted (Fig. 3D).

## 5. Case 3

A 28-year-old multiparous woman was referred to our hospital after experiencing persistent and severe lower abdominal pain for 1 day without any fetal movement. The pain was frequent exacerbation, and was associated with nausea and vomiting. She was at 20 weeks of gestation and multiple prenatal cares all showed no abnormalities. Obstetric history was a normal spontaneous vaginal delivery 4 years ago. Moreover, she had an intrauterine device inserted in 2015 and removed in 2017. A physical examination revealed a normal body temperature, a pulse rate of 60 beats per minute, and a blood pressure of 110/64 millimeters of mercury. The abdomen was tenderness and rebound pain but shifting dullness sign was negative. A significant increase in white blood cell counts and C-reactive protein was observed in laboratory tests. Abdominal ultrasound showed that the shape of the uterine was irregular, the placental blood flow extended to the outside of the uterus, the fetus was in the abdominal cavity, and no heartbeat sign was detected (Fig. 3A and B). Emergency exploratory laparotomy was carried out; we found the shapes of the uterine were gourd-like and a 6cm ruptured wound was situated at the fundus (Fig. 3C). Severe bleeding occurred when we tried to peel the placenta away from the endometrium; therefore, the patient was subjected to a subtotal hysterectomy and diagnosis of placenta accreta is confirmed through postoperative pathologic examination (Fig. 3D). On the 7th postoperative day, the patient was discharged without complications.

## 6. Discussion and conclusion

Uterine rupture is a complete tear or disruption of the uterine muscles and serosa, resulting in catastrophic maternal hemorrhage and the death of both mother and fetus.^[5]^ The disease occurs primarily during late pregnancy and delivery period,^[6]^ and scars resulting from prior uterine surgery(i.e., Cesarean section or myomectomy) remain the foremost risk factor for uterine rupture.^[7]^ The prevalence rate of uterine rupture during pregnancy is 0.05%^[8]^; nevertheless, this number drops to only about 0.005% for scarless pregnant women.^[9]^ Preterm LSCSs, prior wound infections, atypical fetal presentation, advanced maternal age, numerous pregnancies, patients who underwent dilatation and curettage, etc, are some of the other risk factors that are known.^[10,11]^ Monitoring and diagnosis have become difficult due to the diverse clinical presentations of different patients.

First-trimester uterine rupture without scarring is extremely rare,^[12]^ and associated with catastrophic outcomes,^[13]^ which is prone to diagnostic dilemmas. A spontaneous rupture of the unscarred uterus occurred during an early pregnancy reported in this paper. In this case, the exact etiology of uterine rupture is unknown, but it may be caused by an inherited myometrial weakness caused by collagen disorder, which leads to the invasion of trophoblast into the site of previous thinning of the uterine wall and perforation. Our case was once thought to be misdiagnosed as liver rupture; when the diagnosis was clear, laparoscopy was converted to laparotomy, the pregnancy was removed, and the uterine disruption was repaired.

Postoperative pathology revealed placenta increta in 2 further patients. The term “placenta accreta” refers to the abnormal invasion of placental villi into the myometrium caused by a deficient decidua basalis.^[14]^ Until now, the etiology is still unknown with certainty. Depending on the depth of villi invasion placenta accreta is known as “accreta,” “increta” and “percreta,” respectively, which progressively result in serious complications.^[15]^ The common etiologic risk factors were: dilatation and curettage, previous CD, history of retained placenta, uterine anomalies, in vitro fertilization, et al Coincidentally, all 2 women had an operation history of the uterine cavity.

Doctors need to be adept at differential diagnosis in order to treat uterine rupture. In a short amount of time, doctors can differentiate between gynecological conditions, such as burst adnexal cysts, chorioamnionitis, and non-gynecological conditions including appendicitis, intestinal perforation, and kidney stones. Most commonly, this condition is characterized by acute abdominal pain, persistent fetal bradycardia, vaginal bleeding, hypovolemic shock, gastrointestinal distress, etc.^[16]^ In addition, several patients did not exhibit any distinct symptoms and the clinician could not find any specific sign on physical examination, especially for incomplete ruptures.

In patients with suspected uterine rupture, imaging examination is crucial. Ultrasound (US) remains the preferred method of diagnosis because it is easy acquisition, affordable, radiation-free, and can be performed in a variety of settings, including in the operating room or at the bedside of a critically ill patient.^[17]^ However, the US is much less sensitive to the depiction of free intraperitoneal gas.^[18]^ Computed tomography provides a broader feld of view making the abdominal organs’ boundaries sharper and more distinct. Simultaneously, it is more sensitive and valid to assess free gas in the abdominal cavity than US.^[19]^ But attributable to the potential hazards of ionizing radiation and contrast agents, patients did not readily accept it. It is more advantageous to use magnetic resonance imaging for the diagnosis of uterine rupture (UR) due to its superior resolution of soft tissues.^[20]^ However, the options in urgent or emergency situations are limited. The preoperative diagnosis is therefore difficult for physicians, and only surgical intervention may provide a definitive diagnosis. Raising the awareness that pregnant women with abdominal pain may be diagnosed with uterine rupture is of great importance to timely diagnosis and correct treatment. It is essential that this initial period is observed in order to ensure the fetus survival and the life of the mother as well.

Different treatments for uterine rupture during pregnancy may vary depending on maternal and neonatal circumstances, although a hysterectomy is the conventional way to save lives. Patients with stable vital signs who seek to preserve their uterus and fertility are candidates for uterine repair. Women with a history of UR may also have an increased risk of UR during subsequent pregnancies; due to this, those patients were recommended cesarean delivery after fetal lung maturity was documented. In any case, uterine rupture represents an indication for an immediate cesarean section, which should be performed no later than 25 minutes after the first signs of uterine rupture appear. A timely diagnosis of uterine rupture accompanied by the commencement of supportive and surgical care can considerably improve prognosis. Regardless of gestational age and absence of recognized risk factors, all obstetric patients with hemodynamic instability or bleeding (including first-trimester or a previous uterine scar) should be evaluated for uterine rupture. A host of previously unknown risk factors must be considered in patient counseling and clinical practice, including the gestational age, socio-economic status, geographic location, number of previous deliveries, and type of labor.^[13]^ To date, there are no guidelines that are available to guide pregnant women with pregnancy complications in order to prevent the occurrence of UR. Physicians will remain committed to improving the diagnosis and treatment of this disease and to preventing potentially devastating complications, thereby improving the health of mothers and babies.

By diagnosing and intervening promptly in cases of uterine rupture, maternal and fetal outcomes were optimized. Due to the rarity of the disease, its generic clinical presentation, and the absence of primary risk factors, diagnostic challenges exist. As a result, clinical diagnosis is often challenging. Nevertheless, prompt diagnosis and differential diagnosis are critical factors in treating this dangerous disease. So, physicians who are capable of identifying the different risk factors for uterine rupture will be able to diagnose and manage the condition more promptly. Moreover, the training and cultivation of this ability should be popularized by doctors. Any patient with hemoperitoneum should have uterine rupture considered in their differential diagnosis, regardless of their obstetric history. The patients whose story is told in the case report have signed permission for its publication.

## Acknowledgements

We thank all staff who devoted their time and efforts to the study.

## Author contributions

**Data curation:** Jing-Yao She, Si Chen, Chunyun Liang.

**Validation:** Zheng Zeng.

**Writing – original draft:** Yue Chen, Ying Cao.

**Writing – review & editing:** Pei-Juan Wang.
